# Loose–patch recordings reveal differential Kv7-dependent effects on spontaneous excitatory activity in control and epileptic CA1

**DOI:** 10.3389/fneur.2026.1918623

**Published:** 2026-09-11

**Authors:** Ella Marie Nissen, Timo Kirschstein, Astrid Bertsche, Rüdiger Köhling, Denise Franz

**Affiliations:** 1 Oscar Langendorff Institute of Physiology, University Medical Center Rostock, Rostock, Germany; 2 Centre of Transdisciplinary Neurosciences Rostock (CTNR), University Medical Center Rostock, Rostock, Germany; 3 Department of Neuropaediatrics, Hospital for Children and Adolescents, University Medicine Greifswald, Greifswald, Germany

**Keywords:** CA1 neuron, focal epilepsy, KCNQ2 potassium channel, KCNQ3 potassium channel, loose-patch recording

## Abstract

**Introduction:**

The non-inactivating potassium channels Kv7.2 and Kv7.3 contribute to the neuronal resting membrane potential and regulate neuronal excitability. Previous studies have indicated a transcriptional downregulation of these channels in temporal lobe epilepsy, both in patients and in the pilocarpine-induced animal model. However, the functional consequences of this alteration for synaptic activity have not yet been fully elucidated.

**Methods:**

To investigate the role of Kv7 channels in epileptic tissue, we used the pilocarpine model of temporal lobe epilepsy. We performed loose-patch clamp recordings from the perisomatic region of CA1 pyramidal neurons and triggered putative spontaneous excitatory postsynaptic currents (EPSCs) with 5 μM gabazine and 6 mM extracellular K^+^. The effects of the selective Kv7 channel blocker XE991 on EPSC frequency, amplitude, and waveform kinetics (rise time, half-width, decay time) were compared between control and epileptic tissue.

**Results:**

XE991 produced opposing, non-significant within-cell trends in EPSC frequency (decrease in control vs. increase in epileptic tissue), while the absolute EPSC frequency under XE991 differed significantly between groups across all responsive cells (*p* = 0.006). Moreover, XE991 was associated with a trend toward larger EPSC amplitudes and prolonged waveform kinetics, an effect that was more consistently observed in control tissue than in epileptic tissue.

**Discussion/Conclusion:**

These findings demonstrate differential effects of Kv7 channel blockade on spontaneous EPSC frequency and waveform kinetics in epileptic versus control CA1 neurons. The enhanced frequency response, combined with less consistent effects on EPSC waveform duration and amplitude in epileptic tissue, suggests altered Kv7 channel function in the epileptic CA1 region. Together with previous evidence of persistent transcriptional down-regulation of Kv7 channels in the same pilocarpine model, these results support the hypothesis that Kv7 channel dysfunction contributes to network hyperexcitability in temporal lobe epilepsy.

## 1 Introduction

The non-inactivating outward potassium current through Kv7.2 and Kv7.3 channels provides a powerful means of stabilizing the membrane potential due to its voltage dependence, with partial activation at the neuronal resting membrane potential (1, 2). Since mutations of these channels cause a rare neonatal epilepsy (3–5), they have drawn much attention in both clinical and experimental epileptology: On the one hand, Kv7 channel activators such as retigabine are anticonvulsive in both animal models (6–11) and human focal epilepsy (reviewed by (12)). On the other hand, evidence is growing that transcriptional down-regulation of Kv7.2 and Kv7.3 occurs in temporal lobe epilepsy (TLE), demonstrated both in pilocarpine-treated rats (13–15) and in patients (16, 17), while Kv7.5 was preserved in human TLE (18). At least in the pilocarpine model, microRNAs such as 187–3p (19) and 27a−3p (16) were found to be involved in transcriptional down-regulation of potassium channels, including Kv7.2. Hence, Kv7.2/7.3 channel dysfunction is an important target relevant to the pathophysiology of epilepsy.

**Figure 1:**
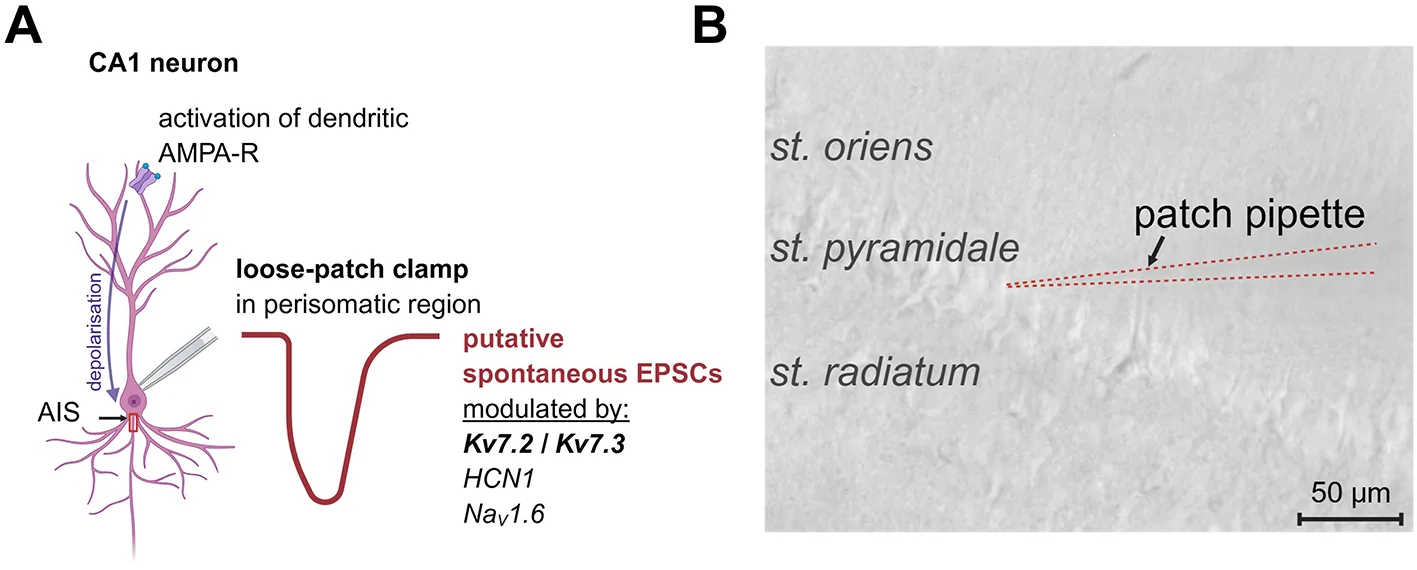
Perisomatic loose-patch recordings on CA1 pyramidal neurons. (**A**) Schematic overview of the generation of the putative spontaneous EPSC in the perisomatic loose-patch configuration, which might be modulated by the Kv7.2/ Kv7.3, HCN1 and Na_v_1.6 ion channels. Here, we investigated the modulation by Kv7.2/ Kv7.3. AIS: axon initial segment, AMPA-R: AMPA receptors (**B**) Light microscopic view of the CA1 region of a horizontal hippocampal slice during loose-patch recording.

With respect to the molecular role of Kv7.2/7.3 channels in epilepsy, much research has focused on the hippocampal CA1 region, where Kv7 inhibition enhanced input resistance and after-depolarising potentials (20–22). This is in line with the documented axosomatic location, including the axonal initial segment (23–25) and axon terminals, at least for Kv7.2 (26, 49). More specifically, data from CA1 neurons even suggested that Kv7 expression was three to five times higher on axons than on dendrites (25). In the entorhinal cortex from pilocarpine-treated epileptic rats, Kv7 channel blockade with linopirdine had no effect in controls, but induced interictal discharges or seizure-like events in epileptic slices (13). Using the same animal model and recording from CA1, we demonstrated that the Kv7 channel blocker XE991 significantly increased the duration and frequency of gabazine-triggered recurrent epileptic discharges in control tissue but not in epileptic tissue (15). Both reports attributed their findings to a critically reduced availability of Kv7.2/7.3 (13, 15). With respect to their role in synaptic transmission, XE991 reduced the input-output relation at Schaffer collateral CA1 synapses only under control conditions, while the paired-pulse ratio was left unaltered in both control and epileptic tissues (15). These findings point to a rather postsynaptic phenotype of Kv7 channel dysfunction in this model. To explore this in more detail, we performed loose-patch clamp recordings in the perisomatic region of CA1 pyramidal neurons to quantify the effects of XE991 on gabazine-triggered putative spontaneous excitatory postsynaptic currents (EPSCs) in the pilocarpine model of epilepsy. We assume that activated AMPA receptors at the dendrites primarily generated these currents. However, with the perisomatic location of our patch pipette, we were also able to investigate the modulation of EPSCs, mainly caused by Kv7.2/7.3 (27), HCN1 (28), and Nav1.6/1.2 (29) channels, which are located on the soma and the axonal initial segment (AIS) (Figure 1A).

## 2 Materials and methods

### 2.1 Pilocarpine-induced status epilepticus

Pilocarpine-induced status epilepticus was performed as described (15). Twenty-two male Wistar rats (33 ± 2 days old, 125 ± 10 g, mean ± SD; Charles River, Sulzfeld, Germany) were pre-treated with N-methylscopolamine (1 mg/kg i.p.; Sigma, Taufkirchen, Germany) to reduce peripheral cholinergic effects (30), 30 min prior to pilocarpine hydrochloride (Pilo; 340 mg/kg i.p.; Sigma). We carefully monitored all pilocarpine-treated rats to detect spontaneous motor seizures with progression into status epilepticus (SE). The onset of SE was assessed when a rat exhibited a stage 4 or 5 seizure (31), followed by continuous convulsions without reaction to sensory stimuli, such as gentle whisker stimulation. Most pilocarpine-treated rats presented motor seizures and eventually developed SE. After 40 min of SE, all convulsions were suppressed by diazepam application (4 mg/kg i.p., repeated as needed). Subsequently, the animals were repeatedly fed with 5% glucose solution for 24 h. Control (Ctrl) rats received saline instead of pilocarpine hydrochloride. Electrophysiological experiments were performed using 11 Pilo and 11 Ctrl rats (1–2 cells per animal), 11 ± 7 weeks after saline or pilocarpine administration, i.e. during the chronically epileptic stage (32).

All animal procedures were carried out according to national and international guidelines on the ethical use of animals (European Council Directive 86/609/EEC) and approved by the “Landesamt für Landwirtschaft, Lebensmittelsicherheit und Fischerei Mecklenburg-Vorpommern” (reference numbers 7221.3–1.1–019/13 and 7221.3–1.1–054/18), and all efforts were made to minimize animal suffering and to reduce the number of animals used.

### 2.2 Slice preparation and loose-patch clamp recording

Following decapitation in deep anesthesia with diethyl ether, brains were rapidly removed and submerged into oxygenated ice-cold sucrose-based dissection solution containing (in mM) 87 NaCl, 75 sucrose, 25 NaHCO_3_, 2.5 KCl, 1.25 NaH_2_PO_4_, 0.5 CaCl_2_, 7 MgCl_2_ and 10 D-glucose (95% O_2_, 5% CO_2_; pH 7.35–7.38; 326–328 mosmol/kg). Unless otherwise indicated, chemicals were purchased from Sigma-Aldrich (Taufkirchen, Germany). Horizontal slices (300 μM thick) were produced using a vibratome (Leica VT 1200 S, Leica Microsystems, Wetzlar, Germany) filled with the above-mentioned sucrose-based dissection solution. Still in this solution, slices were then slowly warmed to 35 °C during 30 min before being inserted into a submerged recording chamber bathed with hyperkalaemic artificial cerebrospinal fluid (ACSF) containing (in mM) 124 NaCl, 26 NaHCO_3_, 6 KCl, 1.25 NaH_2_PO_4_, 2.5 CaCl_2_, 1.3 MgCl_2_, 10 D-glucose (295–305 mosmol/kg, pH = 7.4, 95% O_2_ and 5% CO_2_) and 5 μM gabazine (Tocris, Bristol, United Kingdom) at a flow rate of 1–3 ml/min (35 °C).

CA1 pyramidal cells were visually identified under an upright research microscope (Nikon Eclipse FN1, Tokyo, Japan) equipped with a CCD camera (Retiga 2000 RV, Teledyne Photometrics, Tucson, USA). Neuron viability was visually assessed based on morphology, and a photograph was taken of each measured cell (Figure 1B).

Patch pipettes were made from borosilicate glass (GB150 F-10, Science Products, Hofheim am Taunus, Germany), pulled with a DMZ universal electrode puller (Zeitz, Martinsried, Germany; 3–5 MΩ) and filled with hyperkalaemic ACSF as described above. To record from the immediate vicinity of a CA1 pyramidal cell, the patch electrode was inserted into the ACSF under a positive pressure of no more than 50 mbar and visually approached toward the cell. Once the pipette resistance increased by approximately 0.5 MΩ, we began recording in voltage-clamp mode (HEKA EPC10 and Patchmaster software, Stuttgart, Germany) without applying a holding potential. This increase in pipette resistance was comparable between the two experimental groups (9.1 ± 1.3% in Ctrl versus 7.5 ± 1.2% in Pilo; *P* = 0.259, Mann-Whitney U test). The leak current was not compensated for. The loose-patch configuration was chosen to preserve the intracellular milieu and minimize invasiveness.

**Figure 2:**
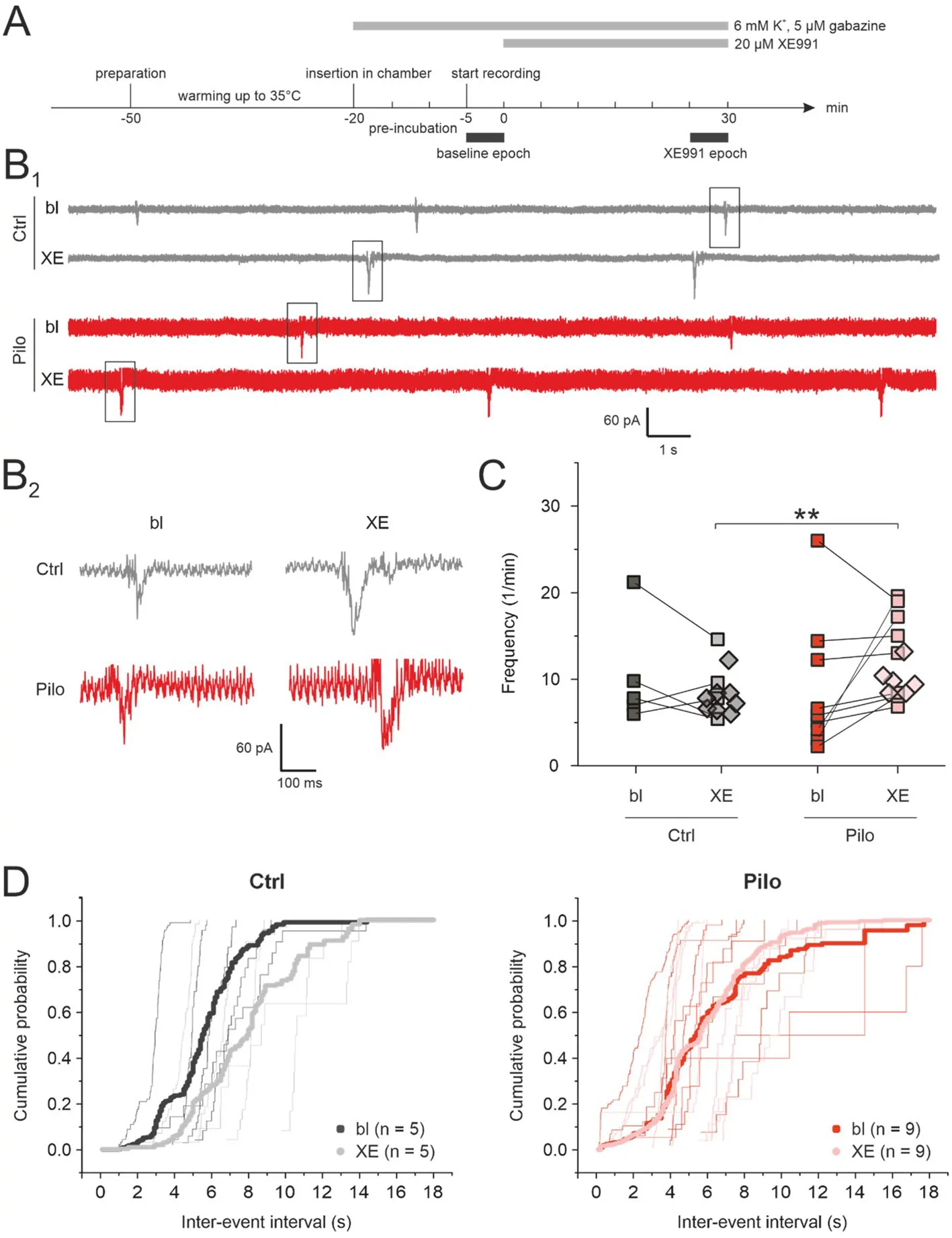
XE991 increases EPSC frequency in epileptic tissue more than in control. **(A)** Timeline of the experimental protocol. **(B)** Sample traces from cell #8 (Ctrl, gray) and #18 (Pilo, red) showing 2–3 EPSCs within a sweep of 20 s, both at baseline (bl) and under XE991 (XE). The boxes in panel B_1_ indicate EPSCs shown enlarged in panel B_2_. **(C)** Scatter plot of EPSC frequencies with connected data points (square symbols) for the paired measurements. Unpaired XE991 results are demonstrated with diamond symbols (^**^p < 0.01 with Mann-Whitney U test). **(D)** Cumulative probabilities for inter-event intervals (ISIs) of paired measurements with thin lines representing individual ISIs and thick lines indicating the average ISI distributions. Further details on the mean values ± SEM and the medians are listed in Supplementary Tables 6, 7.

Following a pre-incubation period of at least 15 min in hyperkalaemic gabazine-containing ACSF, we registered a 5 min epoch consisting of 15 sweeps of 20 s duration at a sample rate of 10 kHz (“baseline epoch” in Figure 2A). Then, XE991 (20 μM) was bath-applied for 30 min, and a further 5 min epoch was registered at the end of this treatment (i.e. from 26th to 30th min after XE991 wash-in, “XE991 epoch” in Figure 2A).

### 2.3 Data analysis

**Figure 3:**
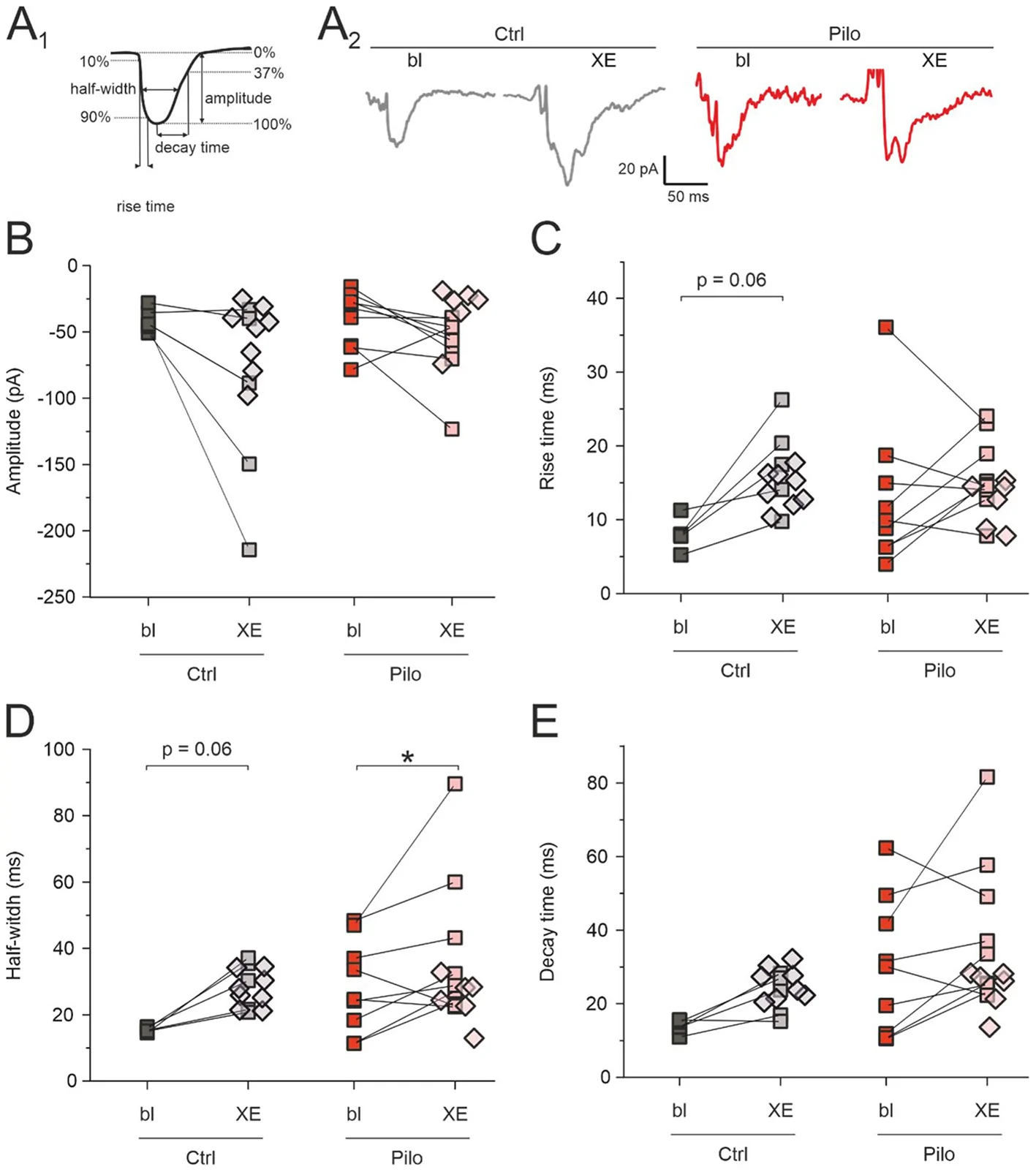
XE991 enlarges and broadens the EPSC in control more than in epileptic tissue. **(A**_**1**_**)** Definition of amplitude and kinetic parameters assessed for each EPSC. **(A**_**2**_**)** Grand averages of EPSCs from cell #8 (Ctrl, gray) and #18 (Pilo, red) during the baseline (bl) epoch (*n* = 49 in Ctrl, *n* = 25 in Pilo) and under XE991 (XE, *n* = 28 in Ctrl, *n* = 34 in Pilo). **(B-E)** Scatter plots with connected data points for the paired measurements and diamond symbols that represent unpaired XE991 results. **(B)** The absolute EPSC amplitudes showed no significant difference between groups and within cells. **(C)** Rise time showed a non-significant trend toward an increase under XE991 (*p* = 0.06, two-sided Wilcoxon signed-rank test). **(D)** The half-width increased in both groups under XE991 application (two-sided Wilcoxon signed-rank test). **(E)** The decay time showed the most notable difference at baseline, but there were no significant within-cell effects. Further details on the mean values ± SEM and the medians are listed in Supplementary Tables 6, 7.

Raw data were first filtered using a 4th-order Bessel filter with a cut-off frequency of 1 kHz, then analyzed using Easy Electrophysiology software (version 2.7.3) to detect spontaneous excitatory postsynaptic currents (EPSCs). Cells that did not respond spontaneously, even in the presence of XE991, were excluded from further analysis. The settings are given in Table 1. With these settings, we ensured that EPSCs were detected only when their amplitude exceeded the noise level by at least a factor of two. The Easy Electrophysiology readout parameters were amplitude, rise time, half-width and decay time for each detected EPSC (Figure 3A1). The rise time was defined as the time between 10% and 90% of the amplitude. The half-width is defined as the full width at half maximum. The decay time was defined as the time between 100% and 37% of the amplitude. In addition, the total number of all EPSCs within the last 5 min of the baseline and XE991-epoch was used to calculate the EPSC frequency, and timestamps of EPSCs were used to determine inter-event intervals (ISI).

**Table 1:** Settings in easy electrophysiology.

Analysis	Parameter/Option	Setting
Baseline		Curve
Threshold		Root mean squared (RMS) of baseline
Amplitude/Decay settings		
	Events analysis options: events fitting	Monoexponential to Decay
	Peak direction	Negative
	Local maximum period	200 ms
	Decay search period	300 ms
16.6-8.6,-13242.3pt	Amplitude threshold	22.7 ± 9.4 pA (9–62 pA; 2 to 7 times of RMS)
Baseline search settings		
	Search period	100 ms

Under baseline conditions, the events in 9 control cells and 12 cells of pilocarpine-treated animals exceeded the predefined detection threshold, allowing quantification of EPSCs. However, individual cell events were only included in the statistical analysis if they met the following cut-off criteria to exclude fast action potential-like events: rise time > 3 ms, half-width > 10 ms, and decay time > 10 ms (in baseline conditions: 5/13 control cells and 9/15 Pilo cells; in XE991 conditions: 13/13 control cells and 15/15 Pilo cells). To perform paired analyses of the XE991 effect, we calculated the absolute difference between the XE991 result and the baseline value (delta change), which was observed in 5 of 13 control cells and 9 of 15 pilocarpine-treated cells. To compare the magnitude of the XE991 effect between experimental groups, the individual delta change values were additionally compared between control and Pilo cells using the Mann-Whitney U test (Supplementary Table 8). Baseline and XE991 measurements obtained from the same cell were analyzed as paired observations, and within-cell changes were assessed using two-sided Wilcoxon signed-rank tests, since the data were not normally distributed (Shapiro-Wilk test). For comparing the control and pilocarpine groups (between-group analysis), the Mann-Whitney U test was used. Statistical evaluation was performed with OriginPro 2018b software. The level of statistical significance was set at *p* < 0.05 and was indicated by asterisks (^*^p < 0.05 or ^**^p < 0.01) in all figures. An overview of all results presented as means ± standard error of the mean (SEM) and medians is provided in Supplementary Tables 6, 7.

The cumulative probabilities for the inter-event intervals (ISIs) were calculated using the empirical cumulative distribution function (ECDF) $F_{j}=\frac{j}{n}$, where (*n*) denotes the number of ISIs. Statistical analyses of ISI distributions were performed at the level of individual cells: ECDFs were generated for each cell that exhibited events under both baseline and XE991 conditions (*n*_control_ = 5; *n*_Pilo_ = 9). To characterize the location and dispersion of the distributions without assuming a specific underlying probability distribution, the median ISI and interquartile range (IQR; Q75–Q25) were calculated directly from the individual ISI values of each cell. Baseline and XE991 conditions were compared within each experimental group using two-sided Wilcoxon signed-rank tests. To assess whether the XE991-induced changes differed between control and pilocarpine-treated cells, within-cell treatment effects for the median ISI and IQR were expressed as log-transformed ratios ($\ln\frac{XE991}{baseline}$) and compared between groups using Mann–Whitney U tests. Because estimates of dispersion are unreliable when based on very few observations, cells with insufficient baseline ISIs (< 10) were excluded.

Weibull cumulative distribution functions were fitted separately to the ISI values for each cell using maximum-likelihood estimation. Model support was assessed using the small-sample-corrected Akaike information criterion (AICc). Among the candidate distributions (exponential, Weibull, gamma, and lognormal), the Weibull distribution provided the most consistently supported common model across cells. Weibull cumulative distribution functions were therefore described according to $F\left(x\right)=1-exp\left[-{\left(\frac{x}{\lambda }\right)}^{k}\right]$. Scale (λ) and shape (k) parameters of the Weibull function were used for descriptive characterization of distribution location and temporal regularity, respectively. However, inference regarding treatment and group effects was primarily based on the distribution-free cell-level median ISI and IQR. For graphical representation of the average ISI distributions, the cumulative probability for each cell was determined over the range 0 to 18 s with a step size of 0.1 s. The mean of each step is represented in the average ISI.

## 3 Results

### 3.1 XE991 effect on EPSC frequency

The present study aimed to compare the effects of Kv7 channel blockade on putative spontaneous excitatory postsynaptic currents (EPSCs) between control and pilocarpine-treated animals. To this end, we first triggered EPSCs by hyperkalaemia (6 mM KCl) and by adding 5 μM gabazine to the bath (Figure 2A). Under these baseline conditions, we obtained EPSCs in 5 of 13 control cells and in 9 of 15 cells from SE-experienced (Pilo) animals. Under XE991, however, spontaneous putative EPSCs were detectable in all cells. Representative recordings of EPSCs from cells #8 (Ctrl) and #18 (Pilo) are depicted in Figure 2B. In addition, the total number of detected EPSCs during baseline and XE991 epochs is given in Supplementary Table 1.

In the paired within-cell analysis, EPSC frequency showed a non-significant decrease of 1.8 ± 1.8/min (5 cells; median change −2.4) in control, and a non-significant increase of 4.0 ± 2.4/min (9 cells; median change +1.8) in Pilo animals (Wilcoxon test, both n.s.; Figure 2C, Supplementary Tables 6, 7). Direct comparison of delta change values between control and Pilo cells revealed no significant between-group difference in the magnitude of the XE991 effect for the frequency (*p* = 0.238, Supplementary Table 8). Across all recorded cells, however, the mean EPSC frequency under XE991 was 8.2 ± 0.7/min (13 cells; median 7.8/min) in controls, but 11.6 ± 1.1/min (15 cells; median 9.8/min) in the Pilo group. This difference was statistically significant (*p* = 0.006; Mann-Whitney U test; Figure 2C, Supplementary Tables 6,7).

To gain more insight into the temporal distribution of EPSCs within the 5-min epochs (baseline and XE991), we constructed cumulative probability plots from the inter-event intervals (ISIs) in a within-cell condition (Figure 2D). The application of XE991 led to a descriptive rightward shift in the average ISI distribution in control cells, indicating longer intervals between consecutive events. In Pilo cells, the average ISI distribution remained comparatively unchanged. Consistent with this observation, the median cell-wise ISI increased non-significantly from 5.8 s at baseline to 8.1 s during XE991 application in controls (*n* = 5; *p* = 0.13). In Pilo cells, after excluding two cells with fewer than 10 baseline ISIs, the median ISI was 4.7 s at baseline and 5.6 s under XE991 (*n* = 7; *p* = 0.22). The relative XE991-induced change in median ISI, expressed as the log-transformed within-cell XE991/baseline ratio, did not differ between control and Pilo cells (*p* = 0.34). Complementary Weibull modeling likewise revealed no statistically significant changes in the following distribution parameters: In control cells, the Weibull scale parameter λ increased non-significantly from 6.3 to 8.3 s (*p* = 0.13), whereas the shape parameter k changed from 7.1 to 8.1 (*p* = 0.44). In Pilo cells, λ remained largely unchanged after XE991 application (5.2 vs 5.1 s; *p* = 0.3), while k increased descriptively from 3.0 to 5.7 (*p* = 0.22). Thus, we found no evidence of temporal clustering in either experimental group following XE991 application. Although none of these changes reached statistical significance, EPSC frequency showed opposite directional trends between the two groups following XE991.

### 3.2 XE991 effect on EPSC amplitude and waveform kinetics

In addition to the XE991 effects on EPSC frequency, we observed that individual EPSC kinetics (see magnified traces in Figure 2B) appeared enlarged and prolonged in response to the Kv7 blocker. To analyse this systematically, we used the Easy Electrophysiology program to fit each EPSC (Figure 3A1). This analysis yielded the EPSC amplitude (in pA) and its waveform kinetics: rise time, half-width, and decay time (all in ms). As shown in Figure 3A2, the averages of EPSCs from cell #8 (Ctrl) and #18 (Pilo) during baseline and under XE991 suggested that both enlargement and prolongation of the EPSCs could be more pronounced in Ctrl than in Pilo cells. In paired recordings, the median EPSC amplitude in control cells doubled by XE991 (−44.1 pA at baseline and −88.5 pA under XE991; *p* = 0.13; Figure 3B, Supplementary Table 6). In Pilo cells, XE991 application raised the median EPSC amplitude to a lesser extent (from −32.5 pA at baseline to −54 pA with XE991), and the difference was not statistically significant either (*p* = 0.1; Figure 3B, Supplementary Table 7). However, a correlation analysis revealed a strong association regarding an amplitude-enhancing effect of XE991 in the control group (correlation coefficient = 0.77; *p* = 0.06; Supplementary Figure 1B). In some control cells, the XE991 application resulted in a markedly increased amplitude (see Supplementary Table 2), which may reflect the configuration of individual ion channels.

After having observed that XE991 increased the EPSC frequency in Pilo rather than in Ctrl cells, but enlarged EPSCs more in Ctrl than in Pilo cells, we focused on waveform kinetic parameters to analyse EPSC morphology: Rise time (Figure 3C), half-width (Figure 3D), and decay time (Figure 3E) increased numerically under XE991 in both groups, although only the increase in half-width in Pilo cells reached statistical significance (*p* = 0.04; Figure 3D). In control cells, all three parameters showed a consistent but non-significant upward trend. With respect to baseline values, the largest numerical difference (though non-significant) was observed for decay time (medians: 30.2 ms for Pilo and 13.6 ms for control; *p* = 0.298, Mann-Whitney U test; Figure 3E). The mean values of the kinetic parameters of all 28 cells at baseline and after application of XE991 are given in Supplementary Tables 3-7; correlation analyses are provided in Supplementary Figure 1C-E. Direct comparison of delta change values between control and Pilo cells revealed no significant between-group differences in the magnitude of the XE991 effect across any parameters (*p* > 0.2; Supplementary Table 8).

Taken together, XE991 was associated with a trend toward larger amplitude and longer EPSC duration. This effect was more consistently observed, though rarely reaching significance, in control cells than in those from pilocarpine-treated animals.

## 4 Discussion

The present study was conducted to explore the effect of the Kv7 channel blocker XE991 on putative spontaneous excitatory postsynaptic currents (EPSCs) in chronically epileptic tissue. In our study, the EPSCs were not evoked by electrical stimulation, but occurred spontaneously in a solution containing 6 mM K^+^ and 5 μM gabazine. One may argue that these conditions triggered rather than truly allowed spontaneous EPSCs. However, combined disinhibition and membrane depolarisation facilitate both asynchronous transmitter release driven by subthreshold calcium transients (33) and synchronous release, as shown for central inhibitory synapses (34). Hence, it is conceivable that the transmitter release depends on the same machinery involved under physiological conditions. Furthermore, despite high K^+^ and gabazine, EPSCs were not detected in about half of the cells (8/13 in control and 6/15 in pilocarpine-treated tissue); no EPSCs were observed unless XE991 was added. This observation implies that our conditions indeed facilitated but did not evoke EPSCs. Electrical stimulation, however, not only evokes large EPSPs but also epileptiform afterpotentials, likely due to recruitment of local networks (15, 35). To study the frequency and amplitude of spontaneous EPSCs, we used disinhibiting conditions and the loose-patch clamp technique. This technique is minimally invasive and entirely preserves intracellular conditions, enabling randomized parallel comparisons between control and Pilo tissue to control for batch effects, as these experiments are independent of the membrane clamp. However, this design cannot, in principle, resolve mono- from polysynaptic contributions or dissociate presynaptic from postsynaptic loci of XE991 action. In contrast to field potential recordings, which predominantly report synaptic currents generated in the dendritic compartment, perisomatic loose-patch recordings are biased toward transmembrane currents arising from the soma and axon initial segment. Consequently, this approach is sensitive to changes in neuronal excitability mediated by ion channels expressed in these compartments, including Kv7.2/7.3, HCN channels, and voltage-gated sodium channels such as Nav1.6.

We found that the absolute EPSC frequency under XE991 was significantly higher in epileptic than in control tissue of the perisomatic region of CA1 pyramidal neurons, driven by a non-significant within-cell increase in Pilo animals, in contrast to a non-significant decrease in controls. Conversely, XE991 increased the amplitude and prolonged the EPSCs, and this effect was notably more consistent in control than in epileptic tissue. Hence, the CA1 epileptic network differentially modulated the XE991 effect on the frequency and waveform kinetics of spontaneous EPSCs. XE991 is a specific blocker of Kv7 channels, with somewhat lower potency against Kv7.1 channels (36, 37). Hippocampal neurons express the isoforms Kv7.2, Kv7.3 and Kv7.5 (38). Opening of heteromers of these isoforms leads to a voltage-dependent K^+^ current with a threshold around −55 mV and a half-maximum activation at around −20 mV (1, 2). Due to their relatively slow activation and deactivation kinetics, with time constants frequently exceeding 100 ms (39), Kv7 channels exert a stabilizing influence on neuronal excitability. Consequently, blockade of Kv7 channels depolarises the resting membrane potential and increases action potential firing (25). From this, it might be hypothesized that XE991 should enhance excitatory network activity. In fact, in our hands, XE991 tended to increase the frequency of EPSCs in epileptic, but not in control tissue. Sun and Kapur (40) reported an increase in miniature EPSC frequency in CA1 pyramidal neurons under conditions that prevented action potential generation, suggesting that Kv7 channel blockade can increase the probability of glutamate release at excitatory synapses. Although the spontaneous EPSCs analyzed in the present study likely include action potential-dependent events, our findings support the notion that Kv7 channels contribute to the control of excitatory synaptic activity and that their blockade enhances EPSC frequency. In contrast to the miniature EPSCs reported by Sun and Kapur (40), spontaneous EPSCs in the present study also exhibited an increase in amplitude following XE991 application. However, since recordings were obtained extracellularly in a perisomatic loose-patch configuration, changes in signal amplitude cannot be straightforwardly interpreted as evidence for increased quantal release or enhanced presynaptic depolarisation. Rather, the recorded events reflect local transmembrane current flows in the perisomatic compartment. In control cells, the statistically non-significant increase in amplitude was accompanied by a parallel, likewise non-significant, prolongation of rise time, half-width, and decay time, whereas in pilocarpine-treated tissue, these parameters were not prolonged. The parallel alteration of all waveform parameters argues against a purely presynaptic mechanism. Instead, it suggests that Kv7 channel blockade modifies the integration and distribution of transmembrane currents within the perisomatic compartment. In contrast, pilocarpine-treated tissue exhibited less consistent changes in EPSC kinetics despite a more pronounced effect on EPSC frequency. This dissociation suggests that Kv7-mediated mechanisms contribute differently to spontaneous excitatory activity in control and epileptic tissue, and raises the question of the extent to which presynaptic Kv7 channels modulate transmitter release. Supported by studies demonstrating that presynaptic Kv7 channels regulate calcium entry and thereby limit transmitter release (41, 42), it is an intriguing aspect for future studies whether this presynaptic Kv7-dependent modulation of transmitter release is probably more prevalent in epileptic than control tissue, which would open up novel therapeutic options. All the same, the divergence between changes in frequency and alterations in waveform kinetics observed in control cells argues against a uniform mechanism of XE991 action and instead suggests that distinct Kv7-dependent processes govern spontaneous excitatory activity and perisomatic signal integration.

The comparatively small change in amplitude observed in pilocarpine-treated animals may be related to the previously reported persistent downregulation of Kv7.2/7.3 channels in this model (15). If Kv7-mediated modulation of perisomatic current integration is already reduced in epileptic tissue, blockade of the remaining channels by XE991 would be expected to exert a smaller additional effect on EPSC waveform kinetics.

As for epilepsy, the XE991-induced facilitation of synaptic transmission explains the proepileptic effects of Kv7.2/7.3 loss-of-function mutations in benign familial neonatal convulsions (3–5). However, clinical phenotype thereof is self-limiting, suggesting that Kv7.2/7.3 dysfunction can be compensated for. This developmental pattern was confirmed by *in vitro* studies: The proepileptic effect of the Kv7 blocker linopirdine was confined to neonatal tissue (43), while this compound failed to induce epileptiform network activity in adult control tissue (44). However, Kv7 channel inhibition facilitated epileptiform activity in combination with carbachol (44) or gabazine and hyperkalaemia (15), demonstrating that functional Kv7.2/7.3 channels were preserved into adulthood. In chronically epileptic tissue, XE991 had previously been found to exert only minor effects on gabazine-induced epileptiform activity, which we interpreted as being consistent with the persistent downregulation of Kv7.2/7.3 channels observed in the pilocarpine model (15). At first glance, the present findings appear to support this interpretation, as XE991 produced comparatively small and inconsistent changes in EPSC waveform kinetics in pilocarpine-treated animals compared with consistent changes in controls. Notably, rise time, half-width, decay time, and amplitude were all more affected in control tissue, suggesting that a substantial fraction of the perisomatic Kv7-dependent modulation is already lost in the epileptic hippocampus. The frequency data, however, require a more nuanced interpretation. XE991 tended to reduce the EPSC frequency in control cells, whereas it tended to increase in pilocarpine-treated animals, although neither within-cell change reached statistical significance (*p* = 0.438 and *p* = 0.074, respectively). Thus, the significant difference between the two experimental groups primarily reflects the relative response to XE991 rather than a uniformly larger absolute increase in the epileptic tissue. Although XE991 did not uniformly increase absolute EPSC frequencies across all recordings, the relative response differed significantly between groups, indicating that Kv7-sensitive mechanisms contribute differently to spontaneous excitatory activity in control and epileptic tissue. Importantly, spontaneous excitatory synaptic activity is frequently reported to be reduced in CA1 pyramidal neurons in the pilocarpine model during the chronic phase, despite the network's overall hyperexcitable state. Reduced excitatory drive and impaired synaptic transmission in CA1 have been described in several studies and are generally considered to reflect seizure-induced network reorganization and neuronal loss (45). Against this background, the low baseline EPSC frequency observed in the pilocarpine group is not unexpected. Taken together, the data suggest that chronic epilepsy does not simply reduce Kv7 function but changes the manner in which Kv7-dependent regulation becomes apparent. Whereas XE991 predominantly altered EPSC waveform characteristics in control tissue, its principal effect in epileptic tissue was a modulation of spontaneous excitatory activity. The finding that XE991 shifted EPSC frequencies toward values observed in control tissue, therefore, suggests that the remaining Kv7-mediated conductance continues to exert a functionally relevant restraint on excitatory activity in the epileptic network. Consequently, the data may suggest a critical dependence of the epileptic hippocampal network on residual Kv7 conductance despite the overall downregulation of Kv7.2/7.3 channels.

Some limitations in our study, however, need to be considered: The molecular attribution of the present pharmacological findings to Kv7.2/7.3 dysfunction relies on prior demonstrations of persistent transcriptional downregulation in the same pilocarpine model (15) rather than on direct molecular measurements in the animals studied here. We did not perform within-cohort molecular validation (qPCR, Western blot, or immunohistochemistry) of Kv7.2/7.3 expression in the present animals. Furthermore, the EPSCs were not pharmacologically tested to determine their origin. Consequently, their glutamatergic origin cannot be definitively confirmed, so we consider them putative EPSCs. Our adjusted signal-to-noise ratio required a minimum EPSC amplitude for detection, potentially leading to the omission of low-amplitude EPSCs. Therefore, we cannot rule out the possibility that silent cells had low-amplitude EPSCs before XE991. Furthermore, fast events may in part reflect action potentials rather than EPSCs, as we did not demonstrate direct modulation of EPSCs. To address this issue, we chose inclusion criteria (rise time > 3 ms, half-width > 10 ms, and decay time > 10 ms) to reduce contamination by action potentials. Moreover, Kv2.1 expressed in HEK293 cells was also blocked by XE991 and linopirdine (39), suggesting a lack of compound specificity. There is at least some evidence linking Kv2.1 reduction and pilocarpine-induced epilepsy (46). Therefore, contributions of Kv2-family channels to the observed effects cannot be completely excluded. In addition, alterations of AMPA receptor function (47) as well as changes in muscarinic receptor expression and signaling (48) have been reported in the pilocarpine model and may have contributed to the observed differences between control and epileptic tissue.

In summary, the present findings suggest that Kv7 channel blockade differentially affects spontaneous excitatory synaptic activity in control and chronically epileptic CA1 networks. On the one hand, EPSC frequency was dampened in controls, but increased in cells from Pilo animals. On the other hand, amplitude and waveform kinetics tended to increase in both experimental groups, with more consistent findings in control tissue. Together with the previously demonstrated downregulation of Kv7.2/7.3 channels in the pilocarpine model (15), these findings suggest that chronic epilepsy does not simply abolish Kv7-dependent regulation but rather changes its functional manifestation. We propose that chronic epilepsy shifts the functional consequences of Kv7 signaling from modulating perisomatic current integration to regulating spontaneous excitatory network activity. Thus, residual Kv7 function may represent a critical stabilizing mechanism in the chronically epileptic hippocampus despite persistent downregulation of Kv7.2/7.3.

## Data Availability

The datasets presented in this study can be found in online repositories. The names of the repository/repositories and accession number(s) can be found in the article.
