# Supplementary material for: Loose–patch recordings reveal differential Kv7-dependent effects on spontaneous excitatory activity in control and epileptic CA1
Source: Front Neurol. 2026 Sep 11;17:1918623. doi: 10.3389/fneur.2026.1918623 (PMC13612331; doi:10.3389/fneur.2026.1918623)
Supplement: Supplementary file 1 [file Table_1.docx]

**Supplementary Figure 1: Correlation analysis of EPSC frequency, amplitude and waveform kinetics under baseline and XE991 conditions.**

Grey dots represent control cells (n = 5) and red dots represent cells from pilocarpine-treated animals (n = 9). (**A**) Correlation of the mean values of EPSC frequency. (**B**) Correlation of the mean values of EPSC amplitude. (**C**) Correlation of the mean values of EPSC of rise time. (**D**) Correlation of the mean values of EPSC of half-width. (**E**) Correlation of the mean values of EPSC Decay time.
