# Supplementary material for: Loose–patch recordings reveal differential Kv7-dependent effects on spontaneous excitatory activity in control and epileptic CA1
Source: Front Neurol. 2026 Sep 11;17:1918623. doi: 10.3389/fneur.2026.1918623 (PMC13612331; doi:10.3389/fneur.2026.1918623)
Supplement: Supplementary file 2 [file Table_2.docx]

**Supplementary Table 1.** Number of spontaneous excitatory postsynaptic currents (EPSCs) in all pyramidal cells during both 5 min recording epochs.

| **Animal** | **Cell#** | **baseline epoch** | **XE991 epoch** |
| --- | --- | --- | --- |
| **Control** | 1 | 0 | 61 |
|  | 2 | 0 | 30 |
|  | 3 | 35 | 48 |
|  | 4 | 0 | 42 |
|  | 5 | 0 | 42 |
|  | 6 | 0 | 33 |
|  | 7 | 0 | 32 |
|  | 8 | 49 | 28 |
|  | 9 | 39 | 27 |
|  | 10 | 0 | 39 |
|  | 11 | 0 | 36 |
|  | 12 | 30 | 39 |
|  | 13 | 106 | 73 |
| **Pilocarpine-treated** | 14 | 0 | 49 |
|  | 15 | 0 | 42 |
|  | 16 | 0 | 42 |
|  | 17 | 0 | 47 |
|  | 18 | 25 | 34 |
|  | 19 | 15 | 98 |
|  | 20 | 72 | 75 |
|  | 21 | 21 | 86 |
|  | 22 | 11 | 40 |
|  | 23 | 0 | 52 |
|  | 24 | 0 | 66 |
|  | 25 | 61 | 65 |
|  | 26 | 29 | 40 |
|  | 27 | 33 | 42 |
|  | 28 | 130 | 95 |

**Supplementary Table 2.** Mean amplitude (in pA) of spontaneous excitatory postsynaptic currents (EPSCs) in all pyramidal cells during both 5 min recording epochs.

| **Animal** | **Cell#** | **baseline epoch** | **XE991 epoch** |
| --- | --- | --- | --- |
| **Control** | 1 |  | -39.38 |
|  | 2 |  | -46.76 |
|  | 3 | -35.37 | -33.11 |
|  | 4 |  | -65.19 |
|  | 5 |  | -79.32 |
|  | 6 |  | -97.85 |
|  | 7 |  | -30.64 |
|  | 8 | -50.83 | -149.74 |
|  | 9 | -28.00 | -39.84 |
|  | 10 |  | -24.86 |
|  | 11 |  | -42.25 |
|  | 12 | -44.14 | -88.50 |
|  | 13 | -45.95 | -214.43 |
| **Pilocarpine-treated** | 14 |  | -34.99 |
|  | 15 |  | -26.25 |
|  | 16 |  | -22.51 |
|  | 17 |  | -74.11 |
|  | 18 | -60.71 | -123.59 |
|  | 19 | -16.08 | -64.10 |
|  | 20 | -21.95 | -53.96 |
|  | 21 | -32.49 | -46.36 |
|  | 22 | -28.29 | -40.92 |
|  | 23 |  | -18.80 |
|  | 24 |  | -25.57 |
|  | 25 | -61.98 | -70.69 |
|  | 26 | -27.46 | -56.61 |
|  | 27 | -39.51 | -39.31 |
|  | 28 | -78.86 | -46.74 |

**Supplementary Table 3.** Mean rise time (in ms) of spontaneous excitatory postsynaptic currents (EPSCs) in all pyramidal cells during both 5 min recording epochs.

| **Animal** | **Cell#** | **baseline epoch** | **XE991 epoch** |
| --- | --- | --- | --- |
| **Control** | 1 |  | 12.02 |
|  | 2 |  | 17.75 |
|  | 3 | 11.27 | 14.10 |
|  | 4 |  | 10.32 |
|  | 5 |  | 15.30 |
|  | 6 |  | 16.22 |
|  | 7 |  | 12.75 |
|  | 8 | 7.94 | 20.38 |
|  | 9 | 8.01 | 26.22 |
|  | 10 |  | 13.54 |
|  | 11 |  | 16.08 |
|  | 12 | 7.76 | 17.47 |
|  | 13 | 5.22 | 9.75 |
| **Pilocarpine-treated** | 14 |  | 14.53 |
|  | 15 |  | 7.80 |
|  | 16 |  | 8.75 |
|  | 17 |  | 12.72 |
|  | 18 | 6.42 | 12.80 |
|  | 19 | 4.02 | 15.22 |
|  | 20 | 6.32 | 14.80 |
|  | 21 | 8.87 | 18.98 |
|  | 22 | 36.10 | 23.04 |
|  | 23 |  | 15.34 |
|  | 24 |  | 14.41 |
|  | 25 | 11.64 | 24.03 |
|  | 26 | 9.96 | 7.86 |
|  | 27 | 15.01 | 14.07 |
|  | 28 | 18.74 | 14.57 |

**Supplementary Table 4.** Mean half-width (in ms) of spontaneous excitatory postsynaptic currents (EPSCs) in all pyramidal cells during both 5 min recording epochs.

| **Animal** | **Cell#** | **baseline epoch** | **XE991 epoch** |
| --- | --- | --- | --- |
| **Control** | 1 |  | 30.35 |
|  | 2 |  | 34.54 |
|  | 3 | 15.31 | 21.63 |
|  | 4 |  | 21.11 |
|  | 5 |  | 25.80 |
|  | 6 |  | 34.18 |
|  | 7 |  | 25.10 |
|  | 8 | 15.90 | 35.41 |
|  | 9 | 14.56 | 37.10 |
|  | 10 |  | 21.37 |
|  | 11 |  | 27.88 |
|  | 12 | 16.34 | 30.26 |
|  | 13 | 15.11 | 20.74 |
| **Pilocarpine-treated** | 14 |  | 24.33 |
|  | 15 |  | 12.91 |
|  | 16 |  | 32.78 |
|  | 17 |  | 22.59 |
|  | 18 | 11.34 | 23.70 |
|  | 19 | 18.27 | 32.34 |
|  | 20 | 11.30 | 27.04 |
|  | 21 | 36.96 | 43.15 |
|  | 22 | 48.27 | 59.92 |
|  | 23 |  | 28.15 |
|  | 24 |  | 28.37 |
|  | 25 | 46.89 | 89.51 |
|  | 26 | 24.04 | 28.78 |
|  | 27 | 24.54 | 22.28 |
|  | 28 | 33.54 | 22.65 |

**Supplementary Table 5.** Mean decay time (in ms) of spontaneous excitatory postsynaptic currents (EPSCs) in all pyramidal cells during both 5 min recording epochs.

| **Animal** | **Cell#** | **baseline epoch** | **XE991 epoch** |
| --- | --- | --- | --- |
| **Control** | 1 |  | 27.64 |
|  | 2 |  | 32.26 |
|  | 3 | 10.96 | 16.89 |
|  | 4 |  | 21.40 |
|  | 5 |  | 22.32 |
|  | 6 |  | 30.43 |
|  | 7 |  | 23.72 |
|  | 8 | 13.59 | 28.28 |
|  | 9 | 13.63 | 23.76 |
|  | 10 |  | 20.46 |
|  | 11 |  | 27.35 |
|  | 12 | 15.04 | 26.96 |
|  | 13 | 15.60 | 15.18 |
| **Pilocarpine-treated** | 14 |  | 28.32 |
|  | 15 |  | 13.65 |
|  | 16 |  | 27.27 |
|  | 17 |  | 21.31 |
|  | 18 | 10.59 | 23.21 |
|  | 19 | 11.91 | 33.56 |
|  | 20 | 10.82 | 25.62 |
|  | 21 | 62.30 | 49.08 |
|  | 22 | 49.45 | 57.66 |
|  | 23 |  | 26.16 |
|  | 24 |  | 28.16 |
|  | 25 | 41.75 | 81.54 |
|  | 26 | 31.57 | 37.08 |
|  | 27 | 19.57 | 25.25 |
|  | 28 | 30.17 | 22.38 |

**Supplementary Table 6.** The summary of results presents the mean values ± SEM and the median (in parentheses) for **control cells** from paired (n=5) and unpaired (n=13) measurements. Statistical analysis reports the p-values: paired measurements were analysed using the two-tailed Wilcoxon signed-rank test, and unpaired measurements using the Mann-Whitney U test.

| ***Event kinetics (control cells)*** | | | | | | |
| --- | --- | --- | --- | --- | --- | --- |
|  | **baseline epoch** (bl) | **Paired statistics** | | **Unpaired statistics** | | |
|  |  | **XE991 epoch** (XE) | **P value** | **XE991 epoch** | **P value** | |
|  |  |  |  |  | **bl** | **XE** |
| Frequency (min^-1^) | 10.4±2.8  (7.8) | 8.6±1.7  (7.8) | 0.438 | 8.2±0.7  (7.8) | 0.298 | 0.006 |
| Amplitude (pA) | -40.9±4.1  (-44.1) | -105.1±34.4  (-88.5) | 0.125 | -73.2±15.3  (-46.8) | 0.699 | 0.273 |
| Rise time (ms) | 8±1  (7.9) | 17.6±2.8  (17.5) | 0.063 | 15.5±1.2  (15.3) | 0.364 | 0.555 |
| Half-width (ms) | 15.4±0.3  (15.3) | 29±3.4  (30.3) | 0.063 | 28.1±1.7  (27.9) | 0.112 | 0.786 |
| Decay time (ms) | 13.8±0.8  (13.6) | 22.2±2.6  (23.8) | 0.125 | 24.4±1.4  (23.8) | 0.298 | 0.17 |
| ***Inter-event intervals (control cells, only paired measurements, n = 5)*** | | | | | | |
|  |  | **Paired statistics** | | | | |
|  | **baseline epoch** | **XE991 epoch** | **P value** | **P value (ln ratio)** | | |
| Median ISI (s) | 5.8 | 8.1 | 0.13 | 0.15 | | |
| IQR (s) | 1.22 | 0.94 | 1 | 0.06 | | |
| Weibull shape k | 7.12 | 8.12 | 0.44 | descriptive | | |
| Weibull scale λ (s) | 6.3 | 8.3 | 0.13 | 0.2 | | |

**Supplementary Table 7.** The summary of results presents the mean values ± SEM and the median (in parentheses) for cells from **pilocarpine-treated** animals in paired (n=9) and unpaired (n=15) measurements. Statistical analysis reports the p-values; paired measurements were analysed using the two-tailed Wilcoxon signed-rank test, and unpaired measurements using the Mann-Whitney U test.

| ***Event kinetics (Pilo cells)*** | | | | | | |
| --- | --- | --- | --- | --- | --- | --- |
|  | **baseline epoch** (bl) | **Paired statistics** | | **Unpaired statistics** | | |
|  |  | **XE991 epoch** (XE) | **P value** | **XE991 epoch** | **P value** | |
|  |  |  |  |  | **bl** | **XE** |
| Frequency (min^-1^) | 8.8±2.5  (5.8) | 12.8±1.7  (13) | 0.074 | 11.6±1.1  (9.8) | 0.298 | 0.006 |
| Amplitude (pA) | -40.8±7.1  (-32.5) | -60.3±8.6  (-54) | 0.1 | -49.6±6.9  (-46.4) | 0.699 | 0.273 |
| Rise time (ms) | 13±3.3  (10) | 16.2±1.7  (14.8) | 0.426 | 14.6±1.2  (14.5) | 0.364 | 0.555 |
| Half-width (ms) | 28.4±4.6  (24.5) | 38.8±7.5  (28.8) | 0.039 | 33.2±4.9  (28.1) | 0.112 | 0.786 |
| Decay time (ms) | 29.8±6.2  (30.2) | 39.5±6.7  (33.6) | 0.129 | 33.4±4.5  (27.3) | 0.298 | 0.17 |
| ***Inter-event intervals (Pilo cells, only paired measurements, n = 7*)*** | | | | | | |
|  |  | **Paired statistics** | | | | |
|  | **baseline epoch** | **XE991 epoch** | **P value** | **P value (ln ratio)** | | |
| Median ISI (s) | 4.7 | 5.6 | 0.22 | 0.34 | | |
| IQR (s) | 1.8 | 0.81 | 0.11 | 0.15 | | |
| Weibull shape k | 3.0 | 5.7 | 0.22 | descriptive | | |
| Weibull scale λ (s) | 5.2 | 5.1 | 0.3 | 0.2 | | |

* Two cells failed due to insufficient baseline ISIs (< 10). However, the plot in Figure 2D demonstrates the cumulative probability of all 9 cells.

**Supplementary Table 8.** Statistical analysis of the delta change values between control (n=5) and Pilo cells (n=9) to analyse the between-group difference in the magnitude of the XE991 effect.

|  | **Median Δ Ctrl** | **Median Δ Pilo** | **P value (Δ Ctrl vs. Δ Pilo)** |
| --- | --- | --- | --- |
| Frequency (min^-1^) | -3.3 | 1.8 | 0.238 |
| Amplitude (pA) | -44.4 | -13.9 | 0.518 |
| Rise time (ms) | 9.7 | 6.4 | 0.24 |
| Half-width (ms) | 13.9 | 11.7 | 0.438 |
| Decay time (ms) | 10.1 | 8.2 | 1 |
